# Microbial colonisation associated with conventional and self-ligating brackets: a systematic review

**DOI:** 10.1177/14653125211056023

**Published:** 2021-11-27

**Authors:** Nidhi P Parmar, Gabrielle L Thompson, Nikki E Atack, Anthony J Ireland, Martyn Sherriff, Jennifer A Haworth

**Affiliations:** Bristol Dental School, University of Bristol, Bristol, UK

**Keywords:** systematic review, self-ligating bracket, conventional bracket, microbial colonisation, fixed orthodontic appliance, randomised controlled trial

## Abstract

**Background:**

Decalcification and gingivitis caused by plaque accumulation around brackets are common iatrogenic effects of fixed appliances. The influence of conventional versus self-ligating bracket design on microbial colonisation is unknown.

**Objective:**

To assess the levels of microbial colonisation associated with conventional and self-ligating brackets.

**Search sources:**

Three databases were searched for publications from 2009 to 2021.

**Data selection:**

Randomised controlled trials comparing levels of microbial colonisation before and during treatment with conventional and self-ligating brackets were assessed independently and in duplicate.

**Data extraction:**

Data were extracted independently by two authors from the studies that fulfilled the inclusion criteria. Risk of bias assessments were made using the revised Cochrane risk of bias tool for randomized trials. The quality of the included studies was assessed using the Critical Appraisal Skills Programme Checklist.

**Results:**

A total of 11 randomised controlled trials were included in this systematic review. Six of the studies were found to be at low risk of bias and five presented with some concerns. The studies were considered moderate to high quality. Five trials reported no statistically significant difference in microbial colonisation between bracket types. The remaining studies showed mixed results, with some reporting increased colonisation of conventional brackets and others increased colonisation of self-ligating brackets. The heterogeneity of study methods and outcomes precluded meta-analysis.

**Conclusion:**

Of the 11 studies included in this systematic review, five found no differences in colonisation between conventional and self-ligating brackets. The remaining studies showed mixed results. The evidence is inconclusive regarding the association between bracket design and levels of microbial colonisation.

## Introduction

In the UK, the National Health Service (NHS) provides orthodontic treatment to more than 200,000 children and teenagers annually (British Orthodontic Society [BOS], 2021a). Increasing numbers of adult patients are also seeking treatment (BOS, 2021b). Labially placed fixed appliances continue to be the appliance of choice, due to their ability to provide 3D control of tooth movement and improved outcomes (Wiedel and Bondemark, 2015). However, orthodontic treatment is not without risk, with notable examples being an increased risk of white spot lesions developing due to plaque accumulation around the appliance (Gorelick et al., 1982) and increased risk of gingivitis (Ristic et al., 2007). Brackets and archwires provide sites for plaque retention, especially at the bracket–tooth interface and a shift in plaque composition can occur during orthodontic treatment due to the presence of the appliance (Ireland et al., 2014), sometimes irrespective of oral hygiene levels (Alfuriji et al., 2014; Atack et al., 1996).

Bracket design has been proposed as an important factor for plaque adhesion and aggregation (Elkordy et al., 2019), and there are two broad types of brackets commonly used in orthodontics, namely conventional brackets (CB) and self-ligating brackets (SLB). While the former utilise elastomeric or stainless-steel ligatures to secure the archwire within the bracket slot, SLBs have a clip to retain the archwire in the slot (Damon, 1998). The presence of a ligature rather than a clip around CBs may hinder effective plaque removal (van Gastel et al., 2009) when compared with SLBs (Harradine, 2013) and bacteria show higher affinities for elastomeric materials, including ligatures, than stainless steel (Türkkahraman et al., 2005). Conversely, regular replacement of elastomeric modules at review visits may avoid development of stagnant areas for long-term bacterial colonisation. The widespread use of fixed appliances and the increased risk of iatrogenic damage from plaque accumulation around orthodontic brackets means it is important to identify whether bracket type influences microbial colonisation. A recent systematic review reported that there is decreased accumulation of *Streptococcus mutans* associated with SLBs compared to CBs (Longoni et al., 2017). Although *S. mutans* is important in the pathogenesis of decalcification, it is important to consider the whole range of Gram-positive microorganisms, such as other streptococci and lactobacilli, as well as Gram-negative microorganisms implicated in periodontal disease and other non-bacterial microorganisms.

## Objective

The objective of this systematic review was to examine evidence from orthodontic randomised controlled trials (RCTs) and determine whether bracket type (CB vs. SLB) has an effect on microbial colonisation.

## Materials and methods

### Protocol and registration

This systematic review was performed and reported in accordance with the Cochrane Handbook for Systematic Reviews of Interventions (Higgins and Green, 2011) and the PRISMA statement (Moher et al., 2009 ). This systematic review was not registered.

### Eligibility criteria

The studies included in the review were RCTs comparing the effects of CB and SLB on levels of microbial colonisation during fixed appliance treatment. Using the components of the Population-Intervention-Comparison-Outcome-Study (PICOS) design scheme, the inclusion and exclusion criteria applied are outlined in Table 1. Limiting the age of participants was not considered to be important for the inclusion criteria. The sampling method and microbial analysis technique were also not limited.

**Table 1.** Eligibility criteria for included studies.

	Inclusion criteria	Exclusion criteria
Population	Participants with healthy periodontal status and no systemic diseases/medication Currently undergoing treatment with orthodontic fixed appliances	Participants with periodontal disease or systemic diseases Any participant undergoing adjunctive treatment
Intervention	CB vs. SLB	Removable appliances/clear aligners, fixed retainer
Comparison	CB vs. SLB	Removable appliances/clear aligners, fixed retainer
Outcome	Assessment of microbial colonisation	Absence of assessment of microbial colonisation
Study design	Randomised controlled trials Human studies	Animal studies Meta-analysis Cohort studies Case-control Cross-sectional studies Case series, Case reports Ideas, opinions, editorials, anecdotal

CB, conventional bracket; SLB, self-ligating bracket.

### Information sources and literature search

An electronic search was performed by two authors (NP and GT) using three databases (MEDLINE [Ovid], Web of Science and Cochrane Library) with the last search date being 30 January 2021. The search terms (Supplementary files 1–3) were adjusted accordingly for each database and limits applied. Limits included English language, RCTs and trials published from 2009–2021, exclusively. Reference lists of eligible articles or existing systematic reviews were also searched.

### Study selection

After the removal of duplicates, the electronic database search yielded 67 results. Two authors (GT and NP) screened the title/abstracts of all papers, removing those that did not satisfy the PICOS criteria and further papers were excluded as appropriate using the criteria shown in Table 1. Any disagreements were resolved through discussion with a third researcher (JAH), resulting in 15 full-text articles to be assessed.

### Data collection and data items

Two authors (NP and GT) extracted the data independently and in duplicate using predefined forms to document: (1) study design; (2) population characteristics; (3) microbial count before and after the use of intervention versus comparator treatments; (4) assessment methods; and (5) follow-up and outcome measurements.

### Risk of bias in individual trials

To assess the risk of bias of each study, two authors (NP and GT) used the revised Cochrane Risk of Bias (RoB) tool for randomised trials (RoB 2.0) (Sterne et al., 2019). NP and GT independently applied this tool to determine a risk of bias judgement for each RCT and, where necessary, in consultation with a third researcher (JAH).

### Outcomes and data synthesis

Only trials comparing CB and SLB were included in this review. No exclusion criteria were set regarding the method used to place the fixed appliances, the teeth involved, split-mouth design or bonding materials used. There were numerous outcome variables, including detection by polymerase chain reaction (PCR) and other DNA techniques or cultivation on agar. The collection time point of microbial samples was not restricted, allowing short- and long-term results to be collected and compared.

The intention was to perform a meta-analysis, but the methods and reported outcomes of the included studies were variable. The outcomes varied from measuring colony-forming units/mL stimulated saliva to quantification of bacterial loads of individual debonded brackets assessed using chemiluminescence from DNA hybridisation. It was deemed that incorporating a meta-analysis was not meaningful.

### Quality of evidence

The Critical Appraisal Skills programme (CASP, 2018) was implemented to assess the quality of the evidence, as recommended by Irving et al. (2017). Using this tool, two authors (NP and GT), independently and in duplicate, evaluated the validity, precision and significance of the results and their applicability to the target population.

## Results

### Study selection

**Figure 1.**
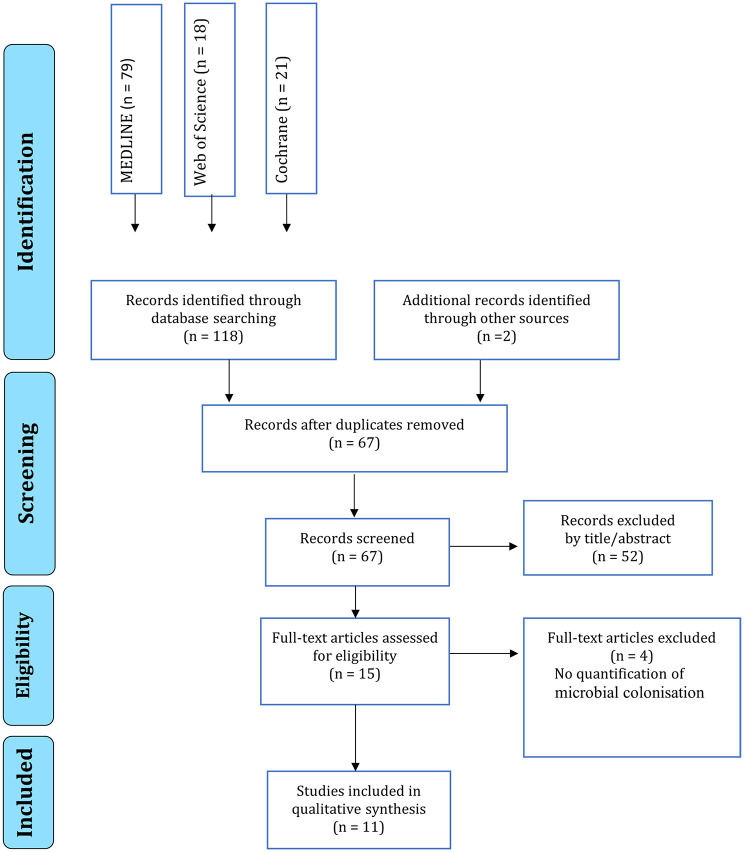
PRISMA flow diagram for study selection process.

The search strategy yielded 67 results and 52 articles were excluded (Supplementary file 4). Fifteen full-text articles remained to be assessed for eligibility. Of these, four more were excluded (Table 2) because, although these studies had appropriate interventions and comparisons, the primary outcome measures were unsuitable. In these studies, periodontal status was recorded but there was no quantification of microbial colonisation. A flowchart (Figure 1) illustrates the search results and selection process.

**Table 2.** Full-text articles excluded with reasons.

Study	Reason for exclusion
Cardoso et al. (2015)	The primary outcome was periodontal status only, with no assessment of microbial colonisation.
Chibber et al. (2018)	The primary outcome was periodontal status only, with no assessment of microbial colonisation.
Folco et al. (2014)	The outcomes were periodontal records and detection of microbial species present. There was no quantification of microbial colonisation.
Kaygisiz et al. (2015)	The outcomes were periodontal records and halitosis, with no assessment of microbial colonisation.

### Study characteristics

The characteristics of the 11 included trials are presented in Table 3. The studies were published between 2009 and 2019. The sample sizes were in the range of 13–60 participants. The mean age of participants in the studies was in the range of 13.3–20.5 years.

**Table 3.** Characteristics of included studies.

Included studies	Study population	Method	Type of bracket used in intervention / control	Outcome
Baka et al. (2013)	20 Split-mouth design	Periodontal measurements before bonding, 1 week after bonding and 3 months after bonding Plaque samples collected from labial surfaces of lateral incisors before bonding and 3 months after bonding Outcome measured using real-time PCR analysis	Damon Q / Roth-equilibrium-2 with stainless-steel ligature	Microbial counts in plaque samples PI PPD BOP
Bergamo et al. (2017)	20 Split-mouth design	1 of each bracket was removed 30 and 60 days after bonding for microbiological analysis Non-stimulated saliva samples collected before bonding, 30 and 60 days after bonding Outcome measured using checkerboard DNA-DNA hybridisation	(1) In-Ovation-R, (2) SmartClip / Gemini	Microbial counts in bracket and saliva samples PI BOP GI
Bergamo et al. (2019)	20 Split-mouth design	Periodontal indices measured 1 of each bracket was removed 30 and 60 days after bonding for microbiological analysis Non-stimulated saliva samples collected before bonding, 30 and 60 days after bonding Outcome measured using checkerboard DNA-DNA hybridisation	(1) In-Ovation-R, (2) SmartClip / Gemini	PI BOP Microbial counts in bracket and saliva samples
Buck et al. (2011)	13 Split-mouth design	4 plaque samples collected per individual from labial and incisal surfaces 1 year after bonding, 1 stimulated saliva sample was also collected per individual Outcome measured using culturing microbial samples on agar plates and ATP bioluminescence	In-Ovation-R / Mini-Ovation	Microbial counts in plaque and saliva samples
Ireland et al. (2014)	24 Split-mouth design Elastomeric ligature placed on SLB on upper lateral incisor, all other teeth had SLBs	Plaque samples collected before bonding, 3 months after bonding, on the day of debond, 3 months after debond and 1 year after debond Samples taken from molars and upper lateral incisors Outcome measured using denaturing gradient gel electrophoresis and 16S rDNA microarray	Damon 2 / Damon 2 with ligature	Plaque scores Microbial counts in plaque samples
Mummolo et al. (2013)	60 20 SLB 20 CB 20 Control group	Stimulated saliva samples collected before bonding and at 3 and 6 months after bonding Outcome measured by culturing microbial samples on agar plates	In-Ovation / Ovation	Microbial counts in saliva samples PI Salivary flow Buffering capacity
Nalcaci et al. (2014)	46 23 SLB 23 CB	Periodontal records, microbial records and halitosis measured before bonding, 1 and 5 weeks after bonding Microbial samples taken from buccal surfaces of all bonded teeth Outcome measured by culturing microbial samples on agar plates	Damon Q / Mini Taurus	Microbial counts in plaque samples PI GI BOP Halitosis
Pandis et al. (2010)	32 16 SLB 16 CB	Whole stimulated saliva collected before treatment and 2–3 months after bonding	In-Ovation-R / Microarch	Microbial counts in saliva samples Simplified plaque index Decayed, missing and filled teeth index
Pejda et al. (2013)	38 19 CB 19 SLB	Supragingival and subgingival plaque samples collected at 18 weeks after bonding Periodontal parameters were recorded before bonding, 6, 12 and 18 weeks after bonding Outcome measured using PCR	Damon 3MX / Sprint	Microbial counts in plaque samples PPD GI BOP Full mouth plaque score
Pellegrini et al. (2009)	14 Split-mouth design	Plaque samples from labial surfaces and saliva samples collected before bonding, 1 and 5 weeks after bonding Outcome measured using ATP bioluminescence	In-Ovation-R / Mini-Ovation	Microbial counts in plaque and saliva samples
Uzuner et al. (2014)	40 20 SLB 20 CB	Periodontal conditions measured, plaque and stimulated saliva samples collected before bonding and 1 month after bonding Outcome measured by culturing microbial samples on agar plates and detected using Dentocult SM™ and LP™ kit (*S. mutans* and *Lactobacillus plantarum* detection kits)	F1000 / Avex MX	Microbial counts in plaque and saliva samples PI GI PPD

BOP, bleeding on probing; CB, conventional bracket; GI, gingival index; PCR, polymerase chain reaction; PI, plaque index; PPD, periodontal probing depth; SLB, self-ligating bracket.

The SLBs used in the studies included Damon Q (Ormco), Damon 2 (Ormco), Damon 3MX (Ormco), In-Ovation R (GAC), Smartclip (3M Unitek) and F1000 (Leone SPA). The CBs included Mini-Ovation (GAC), Ovation (GAC), Roth equilibrium-2 (Dentaurum), Gemini (3M Unitek), Mini Taurus (Rocky Mountain Orthodontics), Sprint (Forestadent), Avex MX (Opal Orthodontics), Microarch (GAC) and a Damon 2 (Ormco) bracket with the use of a ligature.

A variety of outcome measures were reported. All studies quantified microbial colonisation although a wide variety of culture-dependent and culture-independent techniques were used. Seven studies measured additional periodontal parameters such as plaque index, periodontal probing depth, bleeding on probing and gingival index, with one study also measuring salivary flow and buffering capacity. Microbial counts were recorded from plaque or saliva samples; four studies collected plaque samples, two studies collected saliva samples only and five studies collected both plaque and saliva.

The techniques for plaque sampling varied. Supragingival plaque samples were removed from the tooth surface directly adjacent to the brackets using sterilised dental scalers or probes, or a ‘4 pass technique’ was described around the bracket. Plaque was either sampled from all the lateral incisor teeth or from all the bonded teeth. In two studies, one of each bracket type was removed for microbiological analysis (Bergamo et al., 2017, 2019 ). Alternatively, subgingival plaque was collected using sterile paper points. The saliva samples collected were often stimulated, with participants instructed to chew on paraffin wax, but two studies collected non-stimulated saliva. The timepoints of sample collection varied from before bonding, during treatment and up to one-year after debond.

A mixture of culture-dependent and culture-independent techniques were used to analyse the extent of microbial colonisation from the plaque and saliva samples. Molecular techniques predominated, used by seven of the 11 studies. Three studies utilised PCR techniques, two employed adenosine triphosphate (ATP) bioluminescence to measure microbial growth and checkerboard DNA-DNA hybridisation, and denaturing gradient gel electrophoresis and 16S rDNA microarray were also used. Bacterial samples were inoculated on agar plates in four studies.

A split-mouth design was implemented in six studies, with one mimicking a CB by placing an elastomeric ligature on a SLB on an upper lateral incisor (Ireland et al., 2014). The remaining five studies divided the participants into two groups, one receiving CBs and the other SLBs. Only one study had an untreated control group (Mummolo et al., 2013).

### Risk of bias within studies

The risk of bias of the 11 included studies is presented in Table 4. Overall, six of the 11 studies were found to be at low risk of bias (Ireland et al., 2014; Nalcaci et al., 2014; Pandis et al, 2010 ; Pejda et al., 2013; Pellegrini et al., 2009; Uzuner et al., 2014) and five studies presented some concerns (Baka et al., 2013; Bergamo et al., 2017, 2019; Buck et al., 2011; Mummolo et al., 2013). Bias arising from the randomisation process was considered low risk for all 11 studies. Studies implemented different techniques to ensure randomisation. Baseline differences between the intervention groups were homogenous indicating success of randomisation and reducing the risk of selection bias.

**Table 4.** Risk of bias of included trials.

Study	Randomisation process	Deviations from intended outcomes	Missing outcome data	Measurement of the outcome	Selection of the reported results	Overall
Baka et al. (2013)	Low	Some concerns	Low	Some concerns	Low	Some concerns
Bergamo et al. (2017)	Low	Low	Low	Low	Some concerns	Some concerns
Bergamo et al. (2019)	Low	Low	Low	Some concerns	Low	Some concerns
Buck et al. (2011)	Low	Low	Some concerns	Low	Low	Some concerns
Ireland et al. (2014)	Low	Low	Low	Low	Low	Low
Mummolo et al. (2013)	Low	Some concerns	Low	Low	Low	Some concerns
Nalcaci et al. (2014)	Low	Low	Low	Low	Low	Low
Pandis et al. (2010)	Low	Low	Low	Low	Low	Low
Pejda et al. (2013)	Low	Low	Low	Low	Low	Low
Pellegrini et al. (2009)	Low	Low	Low	Low	Low	Low
Uzuner et al. (2014)	Low	Low	Low	Low	Low	Low

Nine studies were considered low risk for bias due to deviations from intended interventions. Two papers had causes for concern (Baka et al., 2013; Mummolo et al., 2013) because outcomes could have been affected by their intervention and analysis methods. Only one loss to follow-up was seen across the studies amounting to an increased risk of attrition bias for that trial (Buck et al., 2011). Bias in measurement of the outcome was low risk in nine studies and of ‘some concern’ in two studies. The trials used appropriate quantitative testing of bacterial loads and kept methods homogenous between intervention groups. Of the 11 studies, 10 adhered to the prespecified analysis plan that was finalised before unblinded outcome data were available (Sterne et al., 2019), thereby reducing risk of reporting bias.

### Results of individual studies and data synthesis

The results of the included studies are presented in Table 5. General trends in the data show a quantitative increase in bacterial loading with both CB and SLB after the initiation of fixed appliance treatment. A range of microorganisms were identified, including *S. mutans*, *Streptococcus sobrinus*, *Lactobacillus casei*, *L. acidophilus*, *Campylobacter rectus*, *Porphyromonas gingivalis*, *Tannerella forsythia*, *Treponema denticola* and *Aggregatibacter actinomycetemcomitans*.

**Table 5.** Study outcomes.

Included studies	Results
Baka et al. (2013)	Differences were not statistically significant between CBs (ligated with stainless steel ligatures) and SLBs (*P* > 0.05). Increases in bacterial populations of *S. mutans*, *S. sobrinus*, *L. casei* and *L. acidophilus* were similar in both groups.
Bergamo et al. (2017)	SLBs were associated with higher red and orange complex bacteria: *P. gingivalis* (*P* = 0.012) *C. rectus* (*P* = 0.011).
Bergamo et al. (2019)	Significant difference in *S. mutans* levels between 3 bracket types at 60 days (*P* = 0.047). InOvation-R SLB had the highest levels of colonisation of *S. mutans* at this timepoint. Levels of salivary microorganisms not compared according to bracket type.
Buck et al. (2011)	No statistical differences in plaque retention between CBs and SLBs after 1 year (*P* > 0.05).
Ireland et al. (2014)	SLBs with an elastomeric ligature showed increased plaque scores compared to SLBs without a ligature. SLBs with an elastomeric ligature showed a greater shift in plaque community composition in the first 3 months of treatment.
Mummolo et al. (2013)	Statistically significant increase in individuals with *S. mutans* salivary counts >10^5^ for the CB group compared to the SLB and control groups during the first 3 months of treatment. Increased *Lactobacillus* species salivary counts in CB group compared to SLB group at 3 months and 6 months.
Nalcaci et al. (2014)	No significant differences in mean counts of *S. mutans* (*P* > 0.05) and lactobacilli (*P* > 0.05) between CBs and SLBs.
Pandis et al. (2010)	No significant difference in salivary *S. mutans* counts between CB and SLB groups (*P* > 0.05).
Pejda et al. (2013)	Statistically significantly higher prevalence of *A. actinomycetemcomitans* in patients with CBs than SLBs. Detection of red complex bacteria (*P. gingivalis*, *Prevotella intermedia*, *T. forsythia* and *T. denticola*) was not statistically significantly different between CB and SLB groups.
Pellegrini et al. (2009)	Decreased levels of total bacteria and oral streptococci in plaque for SLB group compared to CB group at 1 week and 5 weeks after bonding.
Uzuner et al. (2014)	No significant differences in *S. mutans* or *Lactobacillus* salivary or plaque counts between CB and SLB groups 1 month after bonding.

CB, conventional bracket; SLB, self-ligating bracket.

Two studies showed SLBs are associated with increased colonisation by potentially pathogenic microorganisms compared to CBs. SLBs exhibited higher levels of red and orange complex bacterial colonisation compared to CBs (*P. gingivalis: P* = 0.012; *C. rectus: P* = 0.011) (Bergamo et al., 2017). The colours of the complexes represent the pathogenicity of the microorganisms. Purple denotes periodontal health, while orange and red complexes indicate periodontopathogens (Arora et al., 2014). In the study by Bergamo et al. (2019), In-Ovation-R SLBs had the highest levels of colonisation by *S. mutans* at 60 days into treatment.

Three trials concluded that CB encourage increased microbial colonisation compared to SLB (Ireland et al., 2014; Mummolo et al., 2013; Pellegrini et al., 2009) and a study by Pejda et al. (2013) recorded mixed findings. There was a statistically significant increase in *S. mutans* salivary counts >10^5^ for patients with CBs compared to those with SLBs and control groups during the first three months of treatment (Mummolo et al., 2013). The presence of an elastomeric ligature on SLB, simulating CB, was associated with increased plaque scores and a greater shift in plaque community composition in the first three months of treatment compared to SLB without an elastomeric ligature (Ireland et al., 2014). One year after debond, this new plaque microbiome was still identified as being present (Ireland et al., 2014). Decreased levels of total bacteria and oral streptococci in plaque were found in a SLB group compared to a CB group at 1 week and 5 weeks after bonding (Pellegrini et al., 2009). There was a statistically significant higher prevalence of *A. actinomycetemcomitans* in patients with CB than SLB, although in the same study, detection of red complex bacteria (*P. gingivalis*, *T. forsythia* and *T. denticola*) was not significantly different between the two groups (Pejda et al., 2013).

The results from five of the 11 studies were in agreement, detecting no significant differences in levels of microbial colonisation in plaque and/or saliva between CBs and SLBs (Baka et al., 2013; Buck et al., 2011; Nalcaci et al., 2014; Pejda et al., 2013; Uzuner et al., 2014).

### Risk of bias across studies, quality of evidence and additional analyses

The CASP checklist was used to assess the quality of evidence (Table 6). The 11 RCTs were considered to be of moderate to high quality, performing well in all three sections of the checklist.

**Table 6.** Results of CASP checklist questions.

CASP checklist questions	Baka et al. (2013)	Bergamo et al. (2017)	Bergamo et al. (2019)	Buck et al. (2011)	Ireland et al. (2014)	Mummolo et al. (2013)	Nalcaci et al. (2014)	Pandis et al. (2010)	Pejda et al. (2013)	Pellegrini et al. (2009)	Uzuner et al. (2014)
**Section A: Are the results of the trial valid?**											
Did the trial address a clearly focused issue?	Yes	Yes	Yes	Yes	Yes	Yes	Yes	Yes	Yes	Yes	Yes
Was the assignment of patients to treatments randomised?	Yes	Yes	No	Yes	Yes	Yes	Yes	Yes	Yes	Yes	Yes
Were all the patients who entered the trial properly accounted for at its conclusion?	Yes	Yes	Yes	One loss to follow-up	Yes	Yes	Yes	Yes	Yes	Yes	Yes
Were patients, health workers and study personnel ‘blind’ to treatment?	Not disclosed	Not disclosed	Not disclosed	Outcome assessor blind	Not disclosed	Operator and outcome assessor blind	Not disclosed	Operator blind at first sample collection	Outcome assessor blind	Outcome assessor blind	Not disclosed
Were the groups similar at the start of the trial?	Yes	Yes	Yes	Yes	Yes	Yes	Yes	Yes	Yes	Yes	Yes
Aside from the experimental intervention, were the groups treated equally?	Yes	Yes	Yes	Yes	Yes	Yes	Yes	Yes	Yes	Yes	Yes
**Section B: What are the results?**											
How large was the treatment effect? (SLBs vs. CBs)	Not significant (*P* > 0.05)	SLBs significantly higher	SLBs significantly higher (*P* < 0.05)	Not significant (*P* > 0.05)	CBs significantly higher	CBs significantly higher (*P* = 0.001)	Not significant (*P* > 0.05)	Not significant (*P* > 0.05)	Not significant (*P* > 0.05)	CBs significantly higher	Not significant (*P* > 0.05)
How precise was the estimate of the treatment effect?	Unknown (no CI limits)	Unknown (no CI limits)	Unknown (no CI limits)	Precise (95% CI used)	Precise (95% CI used)	Unknown (no CI limits)	Unknown (no CI limits)	Unknown (no CI limits)	Precise (95% CI used)	Precise (95% CI used)	Unknown (no CI limits)
**Section C: Will the results help locally?**											
Can the results be applied to the local population, or in your context?	Probable	Yes	Yes	Yes	Yes	Probable	Yes	Yes	Yes	Yes	Yes
Were all clinically important outcomes considered?	Yes	Yes	Yes	Yes	Yes	Yes	Yes	Yes	Yes	Yes	Yes
Are the benefits worth the harms and costs?	Yes	Yes	Yes	Yes	Yes	Yes	Yes	Yes	Yes	Yes	Yes

CB, conventional bracket; CI, confidence interval; SLB, self-ligating bracket.

The validity of the results was established in section A of the CASP checklist. Only one study had a loss to follow-up (Buck et al., 2011). Operator and participant blinding is difficult to perform clinically because both operator and participant will know which bracket type is being used. However, outcome assessor blinding is possible and was executed in five of the 11 studies. The six papers that did not disclose any blinding had a higher risk of reporting and detection bias, potentially reducing the quality of evidence. The significance of the treatment effect was supported with a *P* value in all studies and the precision of the results was implied by reporting 95% confidence intervals in only four of the studies. The external validity of two studies is likely to be poor; Baka et al. (2013) investigated only male participants and Mummolo et al. (2013) examined 18–23-year-olds, which is less representative of the average treatment age in the general population.

No subgroup analyses, meta-regression analyses or reporting bias analyses were undertaken.

## Discussion

### Summary of evidence

Of the 11 studies selected in this systematic review, five supported the hypothesis that bracket type has no effect on bacterial loading. Other systematic reviews by Nascimento et al. (2014), Yang et al. (2017) and Elkordy et al. (2019) corroborate these findings.

The study by Bergamo et al. (2017) was the only study included in this systematic review reporting that SLBs were associated with a higher incidence of periodontopathogens than CBs, a finding which has been previously reported by van Gastel et al. (2009) and Pithon et al. (2011). Three of the studies reported increased bacterial colonisation in the case of CB (Ireland et al., 2014; Mummolo et al., 2013; Pellegrini et al., 2009). A previous systematic review assessing levels of *S. mutans* colonisation of brackets also reported that SLBs were associated with reduced bacterial colonisation, although the authors cautioned that their conclusions were based on limited evidence (Longoni et al., 2017).

The quality of evidence reported in this review was considered high. All 11 studies performed well against the CASP tool checklist (2018), the use of which has been supported by Irving et al. (2017). Although blinding of outcome assessment was not disclosed in six of the studies, the outcome measurements are objective and therefore less likely to be prone to assessment bias than studies using more subjective techniques.

The RoB 2.0 tool (Sterne et al., 2019) offers a framework for a thorough assessment of risk of bias, and six of the 11 studies included were considered to have a low risk of bias. The heterogeneity of the studies included in this systematic review, both in terms of microbiological techniques and study outcomes, was considered to be too great for data synthesis using meta-analyses (Borenstein et al., 2009).

Plaque retention increases after placement of fixed appliances (Boyd and Baumrind, 1992), which is associated with increased risk of decalcification (Tufekci et al., 2011) and gingival and periodontal changes (van Gastel et al., 2011). Although previous emphasis on the prevalence of *S. mutans* and lactobacilli in the pathogenesis of carious white spot lesions is likely to be oversimplistic (Philip et al., 2018), it is probable that increased plaque accumulation facilitates maturation of the biofilm and recruitment of microorganisms of varied species, including cariogenic species and periodontopathogens. It is important therefore to identify means to reduce plaque accumulation during orthodontic treatment to reduce the chance of iatrogenic damage. Although the studies incorporated in this systematic review do not adopt a “mixed bacterial-ecological approach” (Philip et al., 2018), they still give valuable information about the changes that occur in plaque composition during orthodontic treatment.

This systematic review aimed to examine the evidence as to whether the choice of orthodontic bracket (CB vs. SLB) influences subsequent bacterial biofilm accumulation during orthodontic treatment. Just under half of the studies included found no difference in microbial colonisation between CBs and SLBs. The results of the remaining studies were conflicting, with four favouring SLBs and two favouring CBs. On the basis of this mixed evidence, orthodontists should consider the choice between CB and SLB for reasons other than bacterial colonisation (Elkordy et al., 2019). Regular oral hygiene measures and professional dental visits, regardless of bracket type, are important. These measures aim to prevent development of pathogenic environments leading to enamel decalcification or development of periodontal disease (Ristic et al., 2007). Using equipment such as ‘in-office bacteria tests’ could provide a method for clinicians to monitor bacterial accumulation regularly (Mummolo et al., 2013). Dentists could be incentivised to monitor dietary habits of orthodontic patients in order to maintain an environment that discourages bacterial colonisation (Krupińska-Nanys et al., 2015).

### Strengths and limitations

Excluding all non-RCTs from this systematic review meant that confounding, selection, detection and performance bias were controlled in all 11 studies (Spieth et al., 2016). RCTs exhibit limitations, despite being positioned highly in the hierarchy of evidence (Murad et al., 2016). They require large sample sizes to minimise the random error of chance (Kendall, 2003) and lead to more representative and accurate results. The sample sizes in the studies identified in this review were relatively small, in the range of 13–60 participants, resulting in low statistical power. However, increasing the sample sizes would likely have made the studies more costly and challenging to undertake.

Six of the studies in this review were of split-mouth design, which may be disadvantageous when investigating microbial colonisation. The effects of possible cross-contamination on outcome measures, not only for salivary sampling, but also for in-situ sampling around the brackets, is difficult to quantify. In addition, the effects of clustering in the analysis of data from the split-mouth studies were often not clearly addressed, with only one study (Buck et al., 2011) correlating effects on teeth within individuals.

The majority of participants were adolescents, with the exception of one study investigating 18–23-year-olds (Mummolo et al., 2013), and with the average age of NHS orthodontic patients being 13.4 years (Crosse, 2014) the results of this review can be considered generalisable to a UK NHS orthodontic population (Lavrakas, 2008). One study was less representative as only right-handed male participants were selected (Baka et al., 2013) for inclusion.

A mixture of culture-dependent and culture-independent techniques were presented in the trials included in this review. It is estimated that about 50% of oral bacterial species are resistant to cultivation (Dewhirst et al., 2010) and as such, the use of DNA-based techniques, such as 16S rDNA microarray, real-time PCR and checkerboard DNA-DNA hybridisation, is capable of identifying a different microbial profile compared to culture-dependent techniques. These variable techniques contributed to heterogeneity of the studies within this systematic review.

A limitation of the studies included in this systematic review was the lack of discussion regarding whether any statistically significant differences in microbial colonisation between bracket types, when present, were meaningful clinically. The data presented in the studies also tended to lack confidence intervals, making interpretation of the data more difficult.

A fundamental strength of this systematic review is the focus on microbial colonisation, allowing qualitative analysis and objective reporting of results. However, there was a large number of variables including sample size, participant age, microbiological sampling techniques, point of collection, bracket design, type of ligation, pre-/post-treatment protocols and overall duration of investigation. As a result, data synthesis was limited.

### Recommendations for future research

Overall, this review underpins the necessity for further RCTs assessing the effect of bracket type on microbial colonisation. Future studies should be designed with greater clinical homogeneity and longevity in order to determine if changes in the oral flora are permanent or return to the pre-treatment norm. Only one study investigated this, measuring bacterial loads up to one year after appliance removal (Ireland et al., 2014). Future studies should also aim to link the consequences of changes in microbial colonisation with clinical outcomes, such as incidence of decalcification.

An attempt should also be made to increase blinding and sample sizes, not only to overcome the limitations of RCTs (Mulder et al., 2018), but to allow the inclusion of untreated controls. Finally, it is hoped that future studies in this field will turn to next generation DNA sequencing techniques with less focus on a single pathogen or small group of pathogens, and more emphasis on the whole microbiome (Benn et al., 2018).

## Conclusions

This systematic review identified 11 RCTs comparing microbial colonisation after the placement of either CB or SLB. Just under half the studies included showed no difference in microbial colonisation between CBs and SLBs. The remaining studies reported mixed results. Further work is required to standardise outcomes in clinical trials and to determine the longer-term effects of bracket placement and type on the oral microbiome.

## Supplemental Material

sj-doc-1-joo-10.1177_14653125211056023 – Supplemental material for Microbial colonisation associated with conventional and self-ligating brackets: a systematic review Click here for additional data file. Supplemental material, sj-doc-1-joo-10.1177_14653125211056023 for Microbial colonisation associated with conventional and self-ligating brackets: a systematic review by Nidhi P Parmar, Gabrielle L Thompson, Nikki E Atack, Anthony J Ireland, Martyn Sherriff and Jennifer A Haworth in Journal of Orthodontics

sj-docx-2-joo-10.1177_14653125211056023 – Supplemental material for Microbial colonisation associated with conventional and self-ligating brackets: a systematic review Click here for additional data file. Supplemental material, sj-docx-2-joo-10.1177_14653125211056023 for Microbial colonisation associated with conventional and self-ligating brackets: a systematic review by Nidhi P Parmar, Gabrielle L Thompson, Nikki E Atack, Anthony J Ireland, Martyn Sherriff and Jennifer A Haworth in Journal of Orthodontics

sj-docx-3-joo-10.1177_14653125211056023 – Supplemental material for Microbial colonisation associated with conventional and self-ligating brackets: a systematic review Click here for additional data file. Supplemental material, sj-docx-3-joo-10.1177_14653125211056023 for Microbial colonisation associated with conventional and self-ligating brackets: a systematic review by Nidhi P Parmar, Gabrielle L Thompson, Nikki E Atack, Anthony J Ireland, Martyn Sherriff and Jennifer A Haworth in Journal of Orthodontics

sj-docx-4-joo-10.1177_14653125211056023 – Supplemental material for Microbial colonisation associated with conventional and self-ligating brackets: a systematic review Click here for additional data file. Supplemental material, sj-docx-4-joo-10.1177_14653125211056023 for Microbial colonisation associated with conventional and self-ligating brackets: a systematic review by Nidhi P Parmar, Gabrielle L Thompson, Nikki E Atack, Anthony J Ireland, Martyn Sherriff and Jennifer A Haworth in Journal of Orthodontics

sj-docx-5-joo-10.1177_14653125211056023 – Supplemental material for Microbial colonisation associated with conventional and self-ligating brackets: a systematic review Click here for additional data file. Supplemental material, sj-docx-5-joo-10.1177_14653125211056023 for Microbial colonisation associated with conventional and self-ligating brackets: a systematic review by Nidhi P Parmar, Gabrielle L Thompson, Nikki E Atack, Anthony J Ireland, Martyn Sherriff and Jennifer A Haworth in Journal of Orthodontics
