# Supplementary material for: Microbial colonisation associated with conventional and self-ligating brackets: a systematic review
Source: J Orthod. 2021 Nov 27;49(2):151–62. doi: 10.1177/14653125211056023 (PMC9160783; doi:10.1177/14653125211056023)
Supplement: sj-docx-2-joo-10.1177_14653125211056023 – Supplemental material for Microbial colonisation associated with conventional and self-ligating brackets: a systematic review [file sj-docx-2-joo-10.1177_14653125211056023.docx]

**Supplementary file 1.** MEDLINE search strategy

| Database Used | MEDLINE on Ovid 1946-2021 |  |
| --- | --- | --- |
| Date of Search | 30/01/2021 |  |
| Strategy |  | Results |
| #1 | *Orthodontic Appliances/ae, mi [Adverse Effects, Microbiology] | 682 |
| #2 | Ortho* OR "orthodontic appliance" OR "orthodontic appliances" OR "fixed appliance" OR "fixed appliances" OR "fixed orthodontic appliance" OR "fixed orthodontic appliances" | 414659 |
| #3 | “Oral Microbiota” OR “oral bacteria” OR biofilm OR plaque | 160087 |
| #4 | #2 AND #3 | 3371 |
| #5 | #1 OR #4 | 3919 |
| #6 | Limit #5 to (english language, humans, yr=”2009-2021” and randomised controlled trials) | 160 |
| #7 | Self-ligating OR conventional OR bracket* | 511115 |
| #8 | #6 AND #7 | 79 |
