# Supplementary material for: Microbial colonisation associated with conventional and self-ligating brackets: a systematic review
Source: J Orthod. 2021 Nov 27;49(2):151–62. doi: 10.1177/14653125211056023 (PMC9160783; doi:10.1177/14653125211056023)
Supplement: sj-docx-3-joo-10.1177_14653125211056023 – Supplemental material for Microbial colonisation associated with conventional and self-ligating brackets: a systematic review [file sj-docx-3-joo-10.1177_14653125211056023.docx]

**Supplementary file 2.** Web of Science search strategy

| Database Used | Web of Science Core Collection |  |
| --- | --- | --- |
| Date of Search | 30/01/2021 |  |
| Strategy |  | Results |
| #1 | TS = (Ortho* OR "orthodontic appliance" OR "orthodontic appliances" OR "fixed appliance" OR "fixed appliances" OR "fixed orthodontic appliance" OR "fixed orthodontic appliances") AND language: English Timespan=2009-2021 | 730113 |
| #2 | TS = (“Oral Microbiota” OR oral bacteria OR biofilm OR plaque OR “microbial colonisation”) AND language: English  Timespan=2009-2021 | 186443 |
| #3 | TS = (Self-ligating OR conventional OR bracket*) AND language: English  Timespan=2009-2021 | 800358 |
| #4 | TS = (“randomised controlled trial” OR “random allocation” OR randomisation) AND language: English  Timespan=2009-2021 | 74391 |
| #5 | #1 AND #2 AND #3 AND #4 AND language: English | 18 |
