# Supplementary material for: Microbial colonisation associated with conventional and self-ligating brackets: a systematic review
Source: J Orthod. 2021 Nov 27;49(2):151–62. doi: 10.1177/14653125211056023 (PMC9160783; doi:10.1177/14653125211056023)
Supplement: sj-docx-4-joo-10.1177_14653125211056023 – Supplemental material for Microbial colonisation associated with conventional and self-ligating brackets: a systematic review [file sj-docx-4-joo-10.1177_14653125211056023.docx]

**Supplementary file 3.** Cochrane Library search strategy

| Database Used | Cochrane Central Register of Controlled Trials |  |
| --- | --- | --- |
| Date of Search | 30/01/2021 |  |
| Strategy |  | Results |
| #1 | Ortho* OR "orthodontic appliance" OR "orthodontic appliances" OR "fixed appliance" OR "fixed appliances" OR "fixed orthodontic appliance" OR "fixed orthodontic appliances" | 36715 |
| #2 | “Oral Microbiota” OR oral bacteria OR biofilm OR plaque OR “microbial colonisation” | 18429 |
| #3 | #1 AND #2 | 815 |
| #4 | Self-ligating OR conventional OR bracket* | 60469 |
| #5 | “randomised controlled trial” OR “random allocation” OR randomisation | 107886 |
| #6 | #3 AND #4 AND #5 | 21 |
