# Supplementary material for: Microbial colonisation associated with conventional and self-ligating brackets: a systematic review
Source: J Orthod. 2021 Nov 27;49(2):151–62. doi: 10.1177/14653125211056023 (PMC9160783; doi:10.1177/14653125211056023)
Supplement: sj-docx-5-joo-10.1177_14653125211056023 – Supplemental material for Microbial colonisation associated with conventional and self-ligating brackets: a systematic review [file sj-docx-5-joo-10.1177_14653125211056023.docx]

| Study | Reason for exclusion |
| --- | --- |
| Abbate GM, Caria MP, Montanari P et al. (2015) Periodontal health in teenagers treated with removable aligners and fixed orthodontic appliances. *Journal of Orofacial Orthopedics* *76*(3): 240-250. | Interventions did not include fixed orthodontic appliances |
| Al Ibrahim NS, Tahmassebi JF and Toumba KJ (2010) In vitro and in vivo assessment of newly developed slow-release fluoride glass device. *European Archives of Paediatric Dentistry: Official Journal of the European Academy of Paediatric Dentistry* 11(3): 131-135. | Interventions did not include fixed orthodontic appliances |
| Andrucioli MC, Faria G, Nelson-Filho P et al. (2017) Influence of resin-modified glass ionomer and topical fluoride on levels of Streptococcus mutans in saliva and biofilm adjacent to metallic brackets.*Journal of Applied Oral Science* 25(2): 196-202. | Examined effects of fluoride and glass ionomer on the levels of microbial colonisation during fixed orthodontic therapy only |
| Ashkenazi M, Salem NF, Garon S et al. (2015) Evaluation of Orthodontic and Triple-headed Toothbrushes When Used Alone or in Conjunction with Single-tufted Toothbrush in Patients with Fixed Lingual Orthodontic Appliances. A Randomized Clinical Trial. *New York State Dental Journal* 81(3): 31-37. | Did not report on the levels of microbial colonisation |
| Babacan H, Sokucu O, Marakoglu I et al. (2011) Effect of fixed appliances on oral malodor. *American Journal of Orthodontics and Dentofacial Orthopedics* 139(3): 351-355. | Did not report on the levels of microbial colonisation |
| Beerens MW, Ten Cate JM, Buijs MJ et al. (2018) Long-term remineralizing effect of MI Paste Plus on regression of early caries after orthodontic fixed appliance treatment: a 12-month follow-up randomized controlled trial. *European Journal of Orthodontics*40(5): 457-464. | Did not report on the levels of microbial colonisation |
| Bock NC, Seibold L, Heumann C et al. (2017) Changes in white spot lesions following post-orthodontic weekly application of 1.25 per cent fluoride gel over 6 months-a randomized placebo-controlled clinical trial. Part II: clinical data evaluation. *European Journal of Orthodontics*39(2): 144-152. | Examined methods of oral hygiene control during fixed orthodontic therapy only |
| Bock NC, von Bremen J, Kraft M et al. (2010) Plaque control effectiveness and handling of interdental brushes during multibracket treatment--a randomized clinical trial.*European Journal of Orthodontics* 32(4): 408-413. | Interventions did not include fixed orthodontic appliances |
| Bowen TB, Rinchuse DJ, Zullo T et al. (2015) The influence of text messaging on oral hygiene effectiveness. *The Angle* Orthodontist*85*(4): 543-548. | Interventions did not include fixed orthodontic appliances |
| Charavet C, Lecloux G, Bruwier A et al. (2016) Localized Piezoelectric Alveolar Decortication for Orthodontic Treatment in Adults: A Randomized Controlled Trial.*Journal of Dental Research* 95(9): 1003-1009. | Did not report on the levels of microbial colonisation |
| Cheng HC, Hu HT and Chang YC (2019) Effectiveness of Enzyme Dentifrices on Oral Health in Orthodontic Patients: A Randomized Controlled Trial. *International Journal of Environmental Research and Public Health*16(12): 2243. | Interventions did not include fixed orthodontic appliances |
| Costa MR, da Silva VC, Miqui MN et al. (2010) Effects of ultrasonic, electric, and manual toothbrushes on subgingival plaque composition in orthodontically banded molars.*American Journal of Orthodontics and Dentofacial Orthopedics* 137(2): 229-235. | Examined methods of oral hygiene control during fixed orthodontic therapy only |
| Cunha LDD, Peruzzo DC, Costa LA et al. (2018) Effect of a single-tufted toothbrush on the control of dental biofilm in orthodontic patients: A randomized clinical trial.*International Journal of Dental Hygiene* 16(4): 512-518. | Examined methods of oral hygiene control during fixed orthodontic therapy only |
| Dalessandri D, Dalessandri M, Bonetti S et al. (2012) Effectiveness of an indirect bonding technique in reducing plaque accumulation around braces. The Angle Orthodontist  82(2): 313-318. | Interventions did not include fixed orthodontic appliances |
| Demling A, Elter C, Heidenblut T et al. (2010) Reduction of biofilm on orthodontic brackets with the use of a polytetrafluoroethylene coating.*European Journal of Orthodontics* 32(4): 414-418. | Examined effects of polytetrafluoroethylene coating on the levels of microbial colonisation during fixed orthodontic therapy only |
| Drummond S, Canavarro C, Perinetti G et al. (2012) The monitoring of gingival crevicular fluid volume during orthodontic treatment: a longitudinal randomized split-mouth study. *European Journal of Orthodontics*34(1): 109-113. | Did not report on the levels of microbial colonisation |
| Erbe C, Klukowska M, Tsaknaki I et al. (2013) Efficacy of 3 toothbrush treatments on plaque removal in orthodontic patients assessed with digital plaque imaging: a randomized controlled trial.*American Journal of Orthodontics and Dentofacial Orthopedics* 143(6): 760-766. | Examined methods of oral hygiene control during fixed orthodontic therapy only |
| Esenlik E, Uzer Celik E and Bolat E (2016) Efficacy of a casein phosphopeptide amorphous calcium phosphate (CPP-ACP) paste in preventing white spot lesions in patients with fixed orthodontic appliances: A prospective clinical trial.*European Journal of Paediatric Journal* 17(4): 274-280. | Interventions did not include fixed orthodontic appliances |
| Farhadian N, Bidgoli M, Jafari F et al. (2015) Comparison of Electric Toothbrush, Persica and Chlorhexidine Mouthwashes on Reduction of Gingival Enlargement in Orthodontic Patients: A Randomised Clinical Trial.*Oral Health and Preventative Dentistry* 13(4): 301-307. | Examined methods of oral hygiene control during fixed orthodontic therapy only |
| Farhadian N, Usefi Mashoof R, Khanizadeh S et al. (2016) *Streptococcus mutans* counts in patients wearing removable retainers with silver nanoparticles vs those wearing conventional retainers: A randomized clinical trial. *American Journal of Orthodontics and Dentofacial Orthopedics* 149(2): 155-160 | Interventions did not include fixed orthodontic appliances |
| Fernández-Miñano E, Ortiz C, Vicente, A et al. (2011) Metallic ion content and damage to the DNA in oral mucosa cells of children with fixed orthodontic appliances. *Biometals : An International Journal on the Role of Metal Ions in Biology, Biochemistry, and Medicine* *24*(5): 935–935. | Did not report on the levels of microbial colonisation |
| Fuchslocher Hellemann C, Grade S, Heuer W et al. (2013) Three-dimensional analysis of initial biofilm formation on polytetrafluoroethylene in the oral cavity.*Journal of Orofacial Orthopedics* 74(6): 458-467. | Interventions did not include fixed orthodontic appliances |
| Ghiz MA, Ngan P, Kao E et al. (2009) Effects of sealant and self-etching primer on enamel decalcification. Part II: an in-vivo study.*American Journal of Orthodontics and Dentofacial Orthopedics* 135(2): 206-213. | Interventions did not include fixed orthodontic appliances |
| Ghorbanzadeh R, Pourakbari B and Bahador A (2015) Effects of Baseplates of Orthodontic Appliances with in situ generated Silver Nanoparticles on Cariogenic Bacteria: A Randomized, Double-blind Cross-over Clinical Trial.*The Journal of Contemporary Dental Practice* 16(4): 291-298. | Interventions did not include fixed orthodontic appliances |
| Gomes LK, Sarmento CF, Seabra FR et al. (2012) Randomized clinical controlled trial on the effectiveness of conventional and orthodontic manual toothbrushes.*Brazilian Oral research* 26(4): 360-365. | Examined methods of oral hygiene control during fixed orthodontic therapy only |
| Gómez C, Abellan R and Palma JC (2018) Efficacy of photodynamic therapy vs ultrasonic scaler for preventing gingival inflammation and white spot lesions during orthodontic treatment.*Photodiagnosis and Photodynamic Therapy* 24(1): 377-383. | Examined methods of oral hygiene control during fixed orthodontic therapy only |
| Hammad SM and Knosel M (2016) Efficacy of a new sealant to prevent white spot lesions during fixed orthodontic treatment : A 12-month, single-center, randomized controlled clinical trial.*Journal of Orofacial Orthopedics* 77(6): 439-445. | Did not report on the levels of microbial colonisation |
| Jurisic S, Verzak Z, Jurisic G et al. (2018) Assessment of efficacy of two chlorhexidine mouthrinses on oral hygiene and gingival health in adolescents wearing two types of orthodontic brackets.*International Journal of Dental Hygiene* 16(2): 52-57. | Examined methods of oral hygiene control during fixed orthodontic therapy only |
| Li X, Xu ZR, Tang N et al. (2016) Effect of intervention using a messaging app on compliance and duration of treatment in orthodontic patients.*Clinical Oral Investigations* 20(8): 1849-1859. | Did not report on the levels of microbial colonisation |
| Liptak L, Szabo K, Nagy G et al. (2018) Microbiological Changes and Caries-Preventive Effect of an Innovative Varnish Containing Chlorhexidine in Orthodontic Patients.*Caries Research* 52(4): 272-278. | Examined effects of innovative chlorhexidine varnish on the levels of microbial colonisation during fixed orthodontic therapy only |
| Lombardo L, Ortan YO, Gorgun O et al. (2013) Changes in the oral environment after placement of lingual and labial orthodontic appliances.*Progress in Orthodontics* 14(1): 28. | Examined effects of bracket positioning on the levels of microbial colonisation during fixed orthodontic therapy only |
| Masoud MI, Allarakia R, Alamoudi NM et al. (2015) Long-term clinical and bacterial effects of xylitol on patients with fixed orthodontic appliances.*Progress in Orthodontics* 16(1): 35. | Examined effects of xylitol on the levels of microbial colonisation during fixed orthodontic therapy only |
| Maurya R, Tripathi T and Rai P (2011) New generation of color bonding: a comparative in vitro study.*Indian Journal of Dental Research* 22(5): 733-734. | Interventions did not include fixed orthodontic appliances |
| Mazzoleni S, Bonaldo G, Pontarolo E et al. (2014) Experimental assessment of oral hygiene achieved by children wearing rapid palatal expanders, comparing manual and electric toothbrushes.*International Journal of Dental Hygiene* 12(3): 187-192. | Examined methods of oral hygiene control during fixed orthodontic therapy only |
| Naik SP, Punathil S, Shetty P et al. (2018) Effectiveness of Different Bristle Designs of Toothbrushes and Periodontal Status among Fixed Orthodontic Patients: A Double-blind Crossover Design.*The Journal of Contemporary Dental Practice* 19(2): 150-155. | Examined methods of oral hygiene control during fixed orthodontic therapy only |
| Nammi K, Starke EM, Ou SS et al. (2019) The Effects of Use of a Powered and a Manual Home Oral Hygiene Regimen on Plaque and Gum Health in an Orthodontic Population.*The Journal of Clinical Dentistry* 30(1): 1-8. | Examined methods of oral hygiene control during fixed orthodontic therapy only |
| Nelson-Filho P, Carpio-Horta KO, Andrucioli MC et al. (2012) Molecular detection of *Aggregatibacter actinomycetemcomitans* on metallic brackets by the checkerboard DNA-DNA hybridization technique. *American Journal of Orthodontics and Dentofacial Orthopedics*142(4): 481-486. | Examined the presence and absence of an antimicrobial agent on the levels of microbial colonisation during fixed orthodontic therapy only |
| Nelson-Filho P, Valde RM, Andrucioli MC et al. (2011) Gram-negative periodontal pathogens and bacterial endotoxin in metallic orthodontic brackets with or without an antimicrobial agent: an in-vivo study.*American Journal of Orthodontics and Dentofacial Orthopedics*140(6): 281-287. | Examined methods of oral hygiene control during fixed orthodontic therapy only |
| Niazi FH, Kamran MA, Naseem M (2018) Anti-plaque Efficacy of Herbal Mouthwashes Compared to Synthetic Mouthwashes in Patients Undergoing Orthodontic Treatment: A Randomised Controlled Trial.*Oral Health and Preventative Dentistry* 16(5): 409-416. | Examined methods of oral hygiene control during fixed orthodontic therapy only |
| Nishad A, Sreesan NS, Joy J et al. (2017) Impact of Mouthwashes on Antibacterial Activity of Subjects with Fixed Orthodontic Appliances: A Randomized Clinical Trial.*The Journal of Contemporary Dental Practice* 18(12): 1112-1116. | Examined methods of oral hygiene control during fixed orthodontic therapy only |
| Ousehal L, Lazrak L, Es-Said R et al. (2011) Evaluation of dental plaque control in patients wearing fixed orthodontic appliances: a clinical study.*International Orthodontics* 9(1): 140-155. | Examined methods of oral hygiene control during fixed orthodontic therapy only |
| Paschos E, Kurochkina N, Huth KC et al. (2009) Failure rate of brackets bonded with antimicrobial and fluoride-releasing, self-etching primer and the effect on prevention of enamel demineralization.*American Journal of Orthodontics and Dentofacial Orthopedics*135(5): 613-620. | Did not report on the levels of microbial colonisation |
| Pazzini CA, Marques LS, Ramos-Jorge ML et al. (2012) Longitudinal assessment of periodontal status in patients with nickel allergy treated with conventional and nickel-free braces. *The* *Angle Orthodontist* 82(1): 653–657. | Did not report on the levels of microbial colonisation |
| Peng Y, Wu R, Qu W et al. (2014) Effect of visual method vs plaque disclosure in enhancing oral hygiene in adolescents and young adults: a single-blind randomized controlled trial.*American Journal of Orthodontics and Dentofacial Orthopedics*145(3): 280-286. | Examined methods of oral hygiene control during fixed orthodontic therapy only |
| Pramod S, Kailasam V, Padmanabhan S et al. (2011) Presence of cariogenic streptococci on various bracket materials detected by polymerase chain reaction.*Australian Orthodontic Journal* 27(1): 46-51. | Examined effects of different bracket materials on the levels of microbial colonisation during fixed orthodontic therapy only |
| Pretti H, Barbosa GL, Lages EM et al. (2015) Effect of chlorhexidine varnish on gingival growth in orthodontic patients: a randomized prospective split-mouth study.*Dental Press Journal of Orthodontics* 20(5): 66-71. | Did not report on the levels of microbial colonisation |
| Saruttichart T, Chantarawaratit PO, Leevailoj C et al. (2017) Effectiveness of a motionless ultrasonic toothbrush in reducing plaque and gingival inflammation in patients with fixed orthodontic appliances.*The Angle Orthodontist* 87(2): 279-285. | Examined methods of oral hygiene control during fixed orthodontic therapy only |
| Schlagenhauf U, Kunzelmann KH, Hannig C et al. (2019) Impact of a non-fluoridated microcrystalline hydroxyapatite dentifrice on enamel caries progression in highly caries-susceptible orthodontic patients: A randomized, controlled 6-month trial.*Journal of Investigative and Clinical Dentistry* 10(2): 12399. | Did not report on the levels of microbial colonisation |
| Sfondrini MF, Debiaggi M, Zara F et al. (2012) Influence of lingual bracket position on microbial and periodontal parameters in vivo.*Journal of Applied Oral Science* 20(3): 357-361. | Examined effects of bracket positioning on the levels of microbial colonisation during fixed orthodontic therapy only |
| Silvestrini Biavati A, Gastaldo L, Dessi M et al. (2010) Manual orthodontic vs. oscillating-rotating electric toothbrush in orthodontic patients: a randomised clinical trial.*European Journal of Paediatric Dentistry* 11(4): 200-202. | Examined methods of oral hygiene control during fixed orthodontic therapy only |
| Tufekci E, Pennella DR, Mitchell JC et al. (2014) Efficacy of a fluoride-releasing orthodontic primer in reducing demineralization around brackets: an in-vivo study.*American Journal of Orthodontics and Dentofacial Orthopedics* 146(2): 207-214 | Examined effects of fluoride releasing primer on the levels of microbial colonisation during fixed orthodontic therapy only |
| Yagci A, Seker ED, Demirsoy KK et al. (2019) Do total or partial etching procedures effect the rate of white spot lesion formation? A single-center, randomized, controlled clinical trial. *The* Angle Orthodontist 89(1): 16–24. | Interventions did not include fixed orthodontic appliances |
